# High-Sensitivity Terahertz Refractive Index Sensor in a Multilayered Structure with Graphene

**DOI:** 10.3390/nano10030500

**Published:** 2020-03-10

**Authors:** Jiao Tang, Yunyang Ye, Jiao Xu, Zhiwei Zheng, Xiangliang Jin, Leyong Jiang, Jie Jiang, Yuanjiang Xiang

**Affiliations:** 1 School of Physics and Electronics, Hunan Normal University, Changsha 410081, China; tangjiao@smail.hunnu.edu.cn (J.T.); yeyunyang@smail.hunnu.edu.cn (Y.Y.); xujiao@smail.hunnu.edu.cn (J.X.); zhzhengzhiwei@163.com (Z.Z.); xiangliangjin@163.com (X.J.); 2 Hunan Key Laboratory of Super Microstructure and Ultrafast Process, School of Physics and Electronics, Central South University, Changsha 410083, China; jiangjie@csu.edu.cn; 3 School of Physics and Electronics, Hunan University, Changsha 410082, China

**Keywords:** optical resonance, terahertz sensor, graphene

## Abstract

In this paper, we propose a high-sensitivity optical sensor at terahertz frequencies based on a composite structure containing a one-dimensional photonic crystal (1D PC) coated with a layer of monolayer graphene. Between the 1D PC and the graphene there is a sensing medium. This high-sensitivity phenomenon originates from the excitation of optical resonance between the graphene and the 1D PC. The proposed sensor is highly sensitive to the Fermi energy of graphene, the thickness and refractive index of the sensing medium, and the number of graphene layers. By selecting appropriate parameters, the maximum sensitivity ($407.36^{\circ }/\mathrm{RIU}$) is obtained. We believe the proposed configuration is promising for fabricating graphene-based biosensor- or gas-sensor devices and other related applications in the terahertz band.

## 1. Introduction

An optical sensor, a classical sensor type based on optical principles, can sensitively monitor measured information and convert the information into optical signals or other forms of data according to certain rules [1]. Owing to its advantages (e.g., non-contact and non-destructive measurement, little interference and high sensitivity), the optical sensor supports a wide range of applications in the realm of food safety [2,3], environmental monitoring [4], drug testing [5], medical analysis [6,7], biochemical tests [8,9] and so on. The realization and control means of high-sensitivity optical sensors, especially related optical sensor devices, play a key role in optical measurement and biosensors. In particular, a micro-nano optical sensor, the size of an integrated chip, is the key to information detection and monitoring. Therefore, the realization and testing methods of micro-nano optical sensors have become the center of attention in recent years. For example, scholars have conducted extensive research into carbon nanotubes [10], photonic crystal [11], and graphene/waveguide hybrid structures [12]. In addition, due to the low photon energy in the terahertz band and the distinctive spectral signatures of most biomolecules in the terahertz band, optical sensors working in the terahertz band are also widely used. It is worth mentioning that surface plasmon resonance (SPR) is very sensitive to changes in any boundary environment owing to the boundary propagation of surface plasmon wave. For this reason, SPR-based optical sensors have also become a main focus of attention among researchers. Various high-sensitivity optical sensors have been proposed on the basis of SPR technology [13,14,15]. Recently, graphene has started to play an active role in the realization of high-sensitivity optical sensors due to its excellent optoelectronic properties such as SPR support [16], tunability of optical conductivity [17], broadband [18], etc. In this respect, graphene-based SPR sensors [19,20], hybrid graphene/gold plasmonic fiber-optic sensors [21], mid-infrared plasmonic biosensing with graphene [22], and multi-channel graphene sensors [23], and graphene-based Bloch-like surface wave sensors [24] have been proposed. Recently, Sun et al., reported the application of inorganic/polymer-graphene hybrid gel as a versatile electrochemical platform for an electrochemical capacitor and biosensor [25]. Sun et al., demonstrated the sensitivity enhancement of SPR biosensor based on graphene and barium titanate layers [26]. It can be predicted optimistically that graphene-based or 2D materials-based optical sensors will be one of the most promising application trends [27,28,29]. Although the basic theory of optical sensors is relatively mature, the implementation of optical sensors with a simple structure, high sensitivity and dynamic controllability still remains challenging. Optical sensors with new materials or novel structures and working mechanisms have become the main direction of research in the optical sensor industry. 

We know that the generation of optical resonance has a very positive effect on the realization of high-sensitivity sensor detection. At present, many optical-sensor schemes are mainly realized by the exciting of SPR. This is mainly due to the fact that SPR can produce a very obvious resonance peak, thus creating conditions for sensitive sensor detection. Recently, optical Tamm states (OTSs), a kind of surface wave confined on the contact surface of two different media, have attracted attention in the field of optical sensing [30,31]. It is essentially an interface state. Compared with SPR, OTSs can be excited without a specific angle of incidence, as well as being directly excited by transverse electric (TE) polarization. More importantly, the excitation of OTSs is very sensitive to the change of boundary environment [32]. Therefore, the implementation of optical sensors based on OTSs is very attractive and promising. For example, Zhang et al., proposed a novel concept of a refractive index sensor based on a metal-distributed Bragg reflector [33]. However, the excitation of conventional OTSs is mainly based on a metal-Bragg reflector structure, which does not have dynamic tunability, thus limiting its application in the terahertz band. Graphene not only has excellent tunability and broadband characteristics, but also presents some metal-like properties under certain conditions. This makes it possible to combine graphene and the typical OTS structure to realize a dynamically tunable THz sensor. For example, Ye et al., proposed a graphene-based composite structure to realize a tunable and highly sensitive optical biosensor by exciting OTSs [34]. In this paper, we propose a novel terahertz sensor based on a graphene Bragg reflector composite structure to realize high sensitivity. We find that the high sensitivity of a terahertz sensor is a product of the abnormal reflectance peaks caused by optical resonance. In addition, the tunable conductivity of graphene provides a basis for the design of tunable sensing characteristics in the proposed structure. We believe this electronically-tunable terahertz sensor based on a vertically stacked structure with graphene could offer great potential for applications in the biosensor field.

## 2. Materials and Methods 

**Figure 1:**
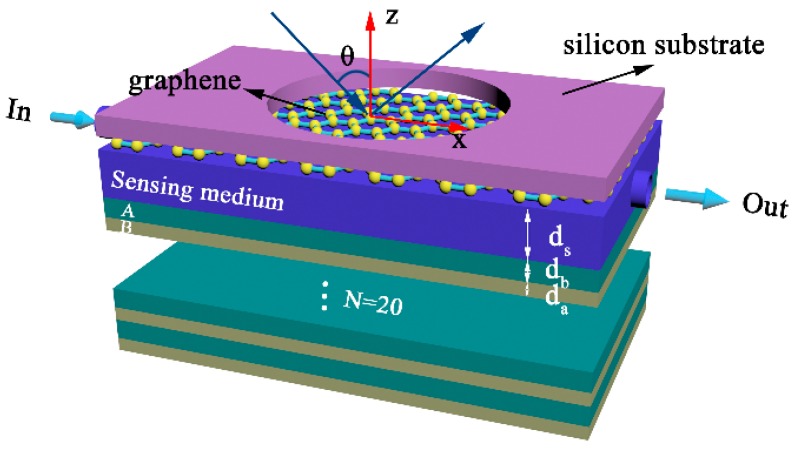
Schematic diagram of the proposed terahertz sensor, where the graphene is coated on the sensing medium and fastened by a fixing device, while a one-dimensional photonic crystal (1D PC) is beneath the sensing medium.

We consider a terahertz sensor by inserting a sensing medium between a polymethylpenten (TPX)/${\mathrm{SiO}}_{2}$ Distributed Bragg Reflector (DBR) and a monolayer graphene film in a vertically stacked structure, with a ${\mathrm{TPX}/\mathrm{SiO}}_{2}$ DBR structure underneath and a monolayer graphene film on the top, as illustrated in Figure 1. Graphene can be transferred to the silicon substrate with holes in consideration of the practically-possible device fabrication. A one-dimensional photonic crystal (1D PC) is formed by alternately stacking dielectric A and dielectric B at a period of N=20. The center wavelength $\lambda_{c}$ is set as 300 μm; the materials of dielectric A and B are selected respectively as ${\mathrm{SiO}}_{2}$ with refractive index $n_{a}=1.46$ and TPX with refractive index $n_{b}=1.9$; the thickness of each 1D PC layer is $d=\lambda_{c}/4n$. The refractive index and original thickness of the sensing medium are, respectively, set as $n_{s}=1.33+\Delta n_{s}$ and $d_{s}=390\;\mu m$; $\Delta n_{s}$ is the change of refractive index of the sensing medium due to the absorption of biomolecules on the surface of graphene. The thickness of graphene, written as $d_{g}=L\times 0.34\;\mathrm{nm}$, is neglected in our calculation; L stands for the number of graphene layers. Here, in order to obtain the physical mechanism more easily and to simplify the calculation, we assume that the refractive indexes of the above materials are dispersionless in the terahertz band. In practice, absorption in the sensing medium cannot be completely avoided, especially in the terahertz band. Therefore, in the next section, we will also briefly discuss the possible influence of the absorption in the sensing layer on the sensitivity performance of the sensor. Besides, the inter-conductivity of graphene is negligible under terahertz band and random phase approximation. According to a Drude-like formula, the conductivity of graphene can be approximately expressed as:

(1) $$\sigma \approx \frac{ie^{2}E_{F}}{\pi \hbar^{2}\left(\omega +i/\tau \right)},$$

where ℏ is the reduced Planck’s constant, $E_{F}$ is the Fermi energy closely related to carrier density ($n_{2D}$), and $E_{F}=\hbar \nu_{F}\sqrt{\pi n_{2D}}$ ($v_{F}\approx 10^{6}m/s$ represents the Fermi velocity of the electron). It creates conditions for us to adjust the conductivity of graphene by controlling the gate voltage. ω is the angular frequency of the incident beam; e and τ represent the elementary electric charge and the relaxation time, respectively. It can be noted that there are some similarities between the above composite structure and the model in Reference [34]. However, the structure of the two is essentially different. In Ref. [34], the incident light needs to pass through the 1D PC with band gap characteristics first, and then contact the sensing medium. The excitation of OTSs has more stringent requirements on the 1D PC. For example, the period of 1D PC cannot be too large, otherwise the incident light cannot penetrate the 1D PC. However, in this work, the incident light first acts on graphene and the sensing medium. At this time, the function of 1D PC is equivalent to the Bragg reflector, and its period needs to be set to a larger value. In addition, the sensing medium in Reference [34] is under the 1D PC, so the influence of the absorption of the sensing medium on the sensitivity of the sensor is much smaller. In our structure, the absorption of the sensing medium has an influence on the sensing performance.

A transfer matrix is applied to calculate the reflectance of the proposed vertically stacked structure [35]. For simplicity, we only consider transverse magnetic (TM) polarization. Then, the transfer matrix between air and the sensing medium can be expressed as:

(2) $$D_{vt}=\frac{1}{2}\left[\begin{array}{cc} 1+\eta_{vs}+\xi_{vs} & 1-\eta_{vs}-\xi_{vs}\\ 1-\eta_{vs}+\xi_{vs} & 1+\eta_{vs}-\xi_{vs} \end{array}\right],$$

where $\eta_{vs}=\varepsilon_{v}k_{sz}/\varepsilon_{s}k_{vz}$ and $\xi_{vs}=\sigma k_{sz}/\varepsilon_{0}\varepsilon_{s}\omega$; $k_{vz}$ and $k_{sz}$ are the wave vector components of light wave propagating in air and sensing medium, respectively. Combined with the propagation matrix of light in dielectric layer P(d)(d is the thickness of the dielectric), the transfer matrix of the whole system can be expressed as: $M=D_{v\to s}P\left(d_{s}\right)D_{s\to a}{\left[P\left(d_{a}\right)D_{a\to b}P\left(d_{b}\right)D_{b\to a}\right]}^{N}$, where N is the period of 1D PC. Thus the reflectance of the structure can be obtained by $R=M_{21}/M_{11}$. In this paper, the reflectance of this structure is strongly sensitive to the variation in the refractive index of the sensing medium. Therefore, the sensitivity of this structure can be expressed as:

(3) $$S=\frac{\Delta \theta }{\Delta n_{s}},$$

where Δθ represents the change of the resonance angle of the proposed structure, and it is caused by the variation in the refractive index of the sensing medium. In addition, the figure of merit (FOM) can be described as: FOM=S⋅DA, where the quality factor (DA) is defined as DA=1/FWHM (full width at half maxima).

## 3. Results and Discussion

**Figure 2:**
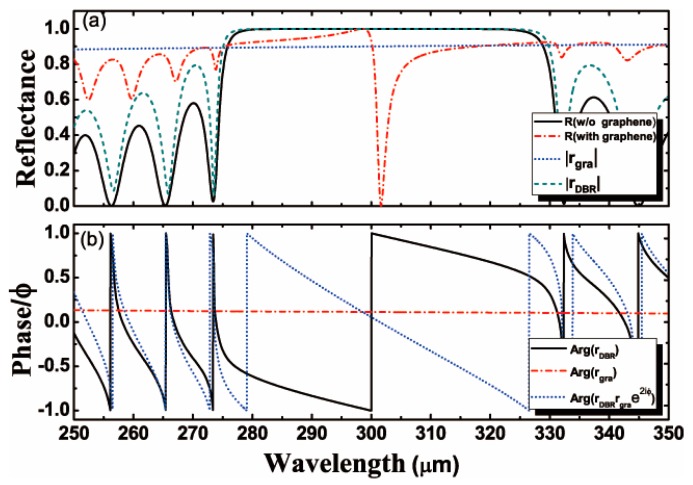
(**a**) The reflectance of the proposed structure (dash dot line), reflectance of the proposed structure in absence of graphene (solid line), reflectance $r_{gra}$ for graphene sensing medium interface (short dot line), and reflectance $r_{DBR}$ for the sensing medium 1D PC interface (short dashed line) as a function of wavelength; (**b**) the phase of $r_{gra}$ (dash dot line), $r_{DBR}$ (soild line), and $r_{DBR}r_{gra}e^{\left(2i\phi \right)}$ (short dotted line) as a function of wavelength.

This section focuses on the sensing characteristics of the proposed structure. In essence, the configuration in Figure 1 can be seen as an asymmetrical cavity consisting of a graphene layer on the left side and a Bragg reflector on the right side, with the sensing medium located inside the cavity. The Fabry–Perot mode can be excited at a certain structural parameter and there is a dip appearing in the reflectance spectrum owing to the excitation of the Fabry–Perot mode. The resonance angle of mode is sensitive to the change of ambient refractive index ($n_{s}$). Therefore, the sensitivity can be calculated by Formula (3). To better illustrate the physics mechanism, the reflectance of the structure with and without graphene are illustrated in Figure 2a. It is found that there is a band gap at a wavelength in the range of 273 μm to 333 μm in the absence of graphene. However, when the sensing medium is coated with graphene, an obvious reflection dip appears at a wavelength of about 300 μm within the band gap. The physical origin of the optical resonance modes observed in Figure 2a can be explained by using the Fabry–Perot mode. However, it is known that graphene is intrinsically a semimetal with some metallic properties under certain conditions. Therefore, the above optical resonance phenomenon can also be explained from the perspective of excitation of OTSs. It is well-known that the excitation of OTSs should satisfy $r_{gra}r_{DBR}e^{\left(2i\phi \right)}=1$, where $r_{DBR}$ is the reflection coefficient of the incident light beam on the sensing medium and the photonic crystal interface, $r_{gra}$ is the reflection coefficient of the incident light beam on the interface of graphene and the sensing medium, and ϕ is the phase change of the light beam impinging at two interfaces of the proposed structure. The resonance frequency position of OTSs excitation can be estimated based on the above formula. Accordingly, $r_{DBR}=-1-0.001i$ and $r_{gra}=2/(1+\eta +\xi )-1$ can be obtained around 300 μm by calculation, where $\eta =\varepsilon_{v}k_{sz}/\varepsilon_{s}k_{vz}=1.335$ and $\xi =\sigma k_{sz}/\varepsilon_{0}\varepsilon_{s}\omega$. When there is no graphene (namely σ=0 and $|r_{gra}|\leq 1$), it will be impossible to excite OTSs. In contrast, the introduction of graphene will bring about ξ≠0 and the formula $r_{gra}r_{DBR}e^{\left(2i\phi \right)}\approx 1$ can be obtained. Furthermore, the excitation of OTSs should also satisfy $Arg(r_{gra}r_{DBR}e^{\left(2i\phi \right)})=0$. As shown in Figure 2b, we obtain $Arg(r_{gra}r_{DBR}e^{\left(2i\phi \right)})\approx 0$ around 300 μm and this result coincides with the dip in Figure 2a.

**Figure 3:**
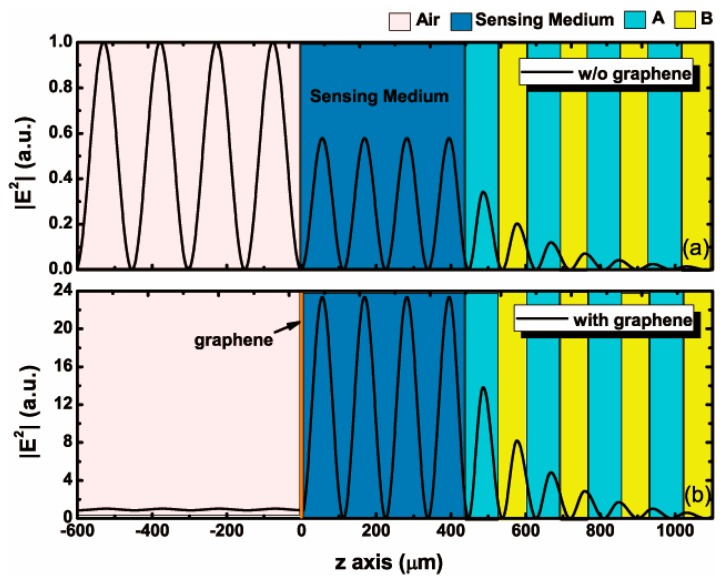
(**a**) The normalized electric field distributions in the proposed structure in absence of monolayer graphene. (**b**) The normalized electric field distributions in the proposed strcture coated with monolayer graphene.

In order to further illustrate the relevance of graphene to the strong excitation of the resonance mode, we cover the sensing medium with monolayer graphene and draw the normalized electric field distributions in Figure 3a. The normalized electric field distributions in the absence of graphene are plotted in Figure 3b for comparison. The position of graphene is set as z=0. It is shown that the light beam impinges on the interface between the graphene and the sensing medium, and the sharp rise of the electric field near the graphene coincides precisely with the abnormal dip of reflectance in Figure 2a. When the light beam penetrates the photonic crystal, the electric field decays rapidly with the increase of PC periods. The local field intensity is enhanced significantly in the structure coated with graphene. These results have proved that graphene plays a positive role in the excitation of resonance mode and the realization of a high sensitivity of the proposed structure. 

**Figure 4:**
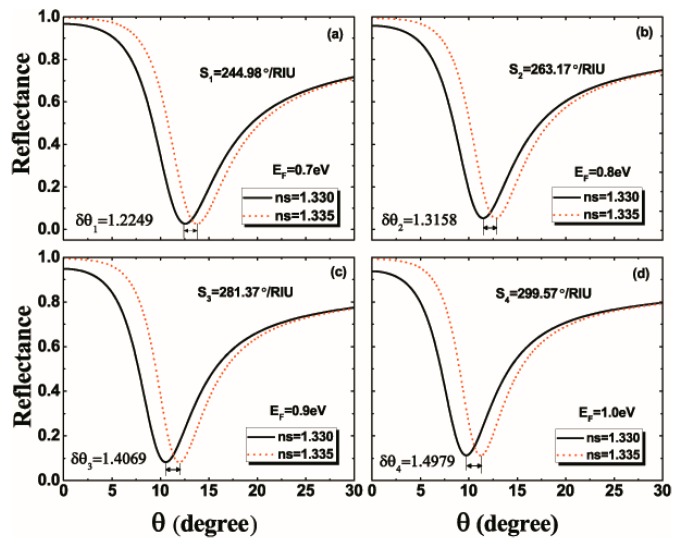
Variations of reflectance with respect to the incident angle under (**a**) $E_{F}=0.7\;\mathrm{eV}$, (**b**) $E_{F}=0.8\;\mathrm{eV}$, (**c**) $E_{F}=0.9\;\mathrm{eV}$, (**d**) $E_{F}=1.0\;\mathrm{eV}$. The relaxation time and number of graphene layers are set as τ=0.5 ps and L=1, respectively. The sensing medium is set as $d_{s}=390\;\mu m$.

To enhance the sensitivity and to expand the measuring range of the proposed terahertz refractive index sensor, we also take the major parameters of graphene, and the thickness and refractive index of the sensing medium into consideration. The variations in the reflectance at different levels of Fermi energy with respect to the incident angle are plotted, as shown in Figure 4. For a sensing medium containing biomacromolecules, we select aqueous solution with a refractive index of 1.33; τ and L are set to be 0.5 ps and 1, respectively. Graphene, in comparison with a metal surface, also shows strong and stable absorption to biomolecules. For the calculation of sensitivity, the change of the refractive index of the sensing medium is assumed to be $\Delta n_{s}=0.005$. The optical properties of graphene are represented by electrical conductivity, and the conductivity of graphene can be regulated by adjusting the Fermi energy and relaxation time. Furthermore, we regulate the Fermi energy of graphene by adjusting the external voltage. The sensitivity of the proposed structure is as high as $299.57^{\circ }/\mathrm{RIU}$ when $E_{F}=1\;\mathrm{eV}$, as shown in Figure 4d. Through research, the optimal Fermi energy of graphene should be 1 eV. It is worth mentioning that the above sensitivity performance is calculated without considering the absorption of the sensing medium. In fact, the influence of the absorption coefficient of the sensing medium on the sensing characteristics also needs to be considered, especially in the terahertz band. Therefore, based on the structure of Figure 1, we further calculate the sensitivity performance of the terahertz refractive index sensor in three cases: (1) when the sensing medium is an aqueous solution, the dielectric function of water can be expressed by a triple Debye function: $\varepsilon_{water}\left(\omega \right)=\sum_{i=1}^{3}\Delta \varepsilon_{i}/\left(1+j\omega \tau_{i}\right)+\varepsilon_{\infty }$, where $\Delta \varepsilon_{1}=69.1,$ $\Delta \varepsilon_{2}=2.01,$ $\Delta \varepsilon_{3}=2.08,$ $\tau_{1}=9.02\;\mathrm{ps},$ $\tau_{2}=0.8\;\mathrm{ps},$ $\tau_{3}=0.05\;\mathrm{ps}$ [36]. It is found that the resonance phenomenon cannot occur obviously because the aqueous solution has a large absorption coefficient in the terahertz band. Therefore, it is difficult to use aqueous solution as sensing medium in terahertz band. (2) We also calculated the sensing characteristics when the sensing medium is a liquid with low absorption coefficient (for example, the absorption of nonpolar solution is generally low in the terahertz band). Take n-propanol as an example in the calculation ($E_{E}=0.8\;\mathrm{eV},\;\tau =0.7\;\mathrm{ps},\;d_{s}=390\;\mu m$, with other parameters having the same values as those in Figure 4) [37]. It is found that the resonance phenomenon can still be realized and the sensitivity of about 400°/RIU can be obtained. (3) Furthermore, when the sensing medium is gas (where the refractive index is close to 1 and absorption can be ignored), the structure in the manuscript can be further developed into a THz gas sensor. In the case of similar structural parameters as before, the resonance phenomenon is very obvious and the sensitivity above 500°/RIU can be obtained. The above results indicate that a high-sensitivity terahertz refractive index sensor based on this structure is feasible.

**Figure 5:**
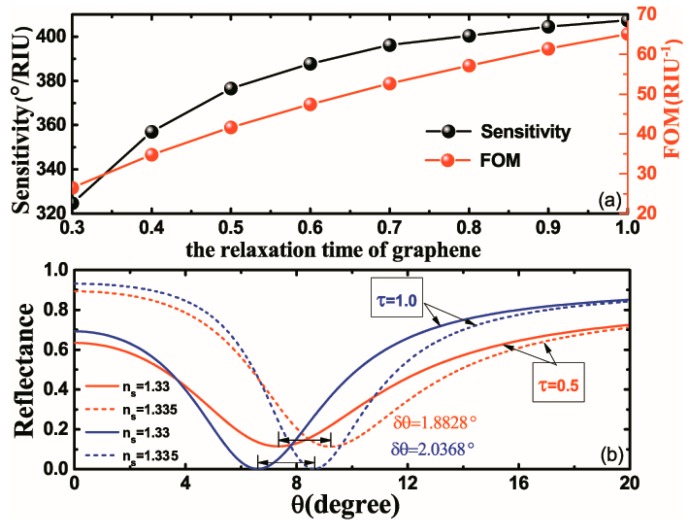
(**a**) The sensitivity and FOM of the proposed structure with respect to the relaxation time of graphene varying from 0.3 ps to 1.0 ps. (**b**) The reflectance as a function of incident angle at τ=0.5 ps and τ=1.0 ps; the Fermi energy is set to $E_{F}=1.0\;\mathrm{eV}$; other parameters are the same as those in Figure 2.

It can be seen from the formula of graphene conductivity that the relaxation time also has an obvious effect on conductivity. However, there are sometimes obstacles to regulating the relaxation time of graphene, because it is difficult to change the relaxation time once the graphene is prepared. Nevertheless, it is necessary to evaluate systematically the effects of relaxation time on the sensing properties of the proposed structure, as shown in Figure 5. The variations of the sensitivity and FOM with respect to the relaxation time of graphene are plotted in Figure 5a. It is found that the narrower the FHWM is, the higher the structural sensitivity would be. Meanwhile, Figure 5b shows the variation of reflectance as a function of the incident angle when τ=1.0 ps and τ=0.5 ps. When τ=1.0 ps, the highest sensitivity of the proposed structure (407.36°/RIU) is obtained. It can be observed that the sensitivity and FOM vary monotonously with the relaxation time, mainly because the increase of relaxation time has a strong influence on the real part of the conductivity but is barely effective for the imaginary part, and this is reflected in narrower and deeper reflectance curves. Consequently, the sensitivity and FOM of the proposed structure are affected. The FHWM of the reflectance at τ=0.5 ps is obviously smaller than that at τ=1.0 ps, thus producing the highest FOM (65 RIU^−1^). In summary, the effect of relaxation time on sensitivity is more significant than that on Fermi energy.

**Figure 6:**
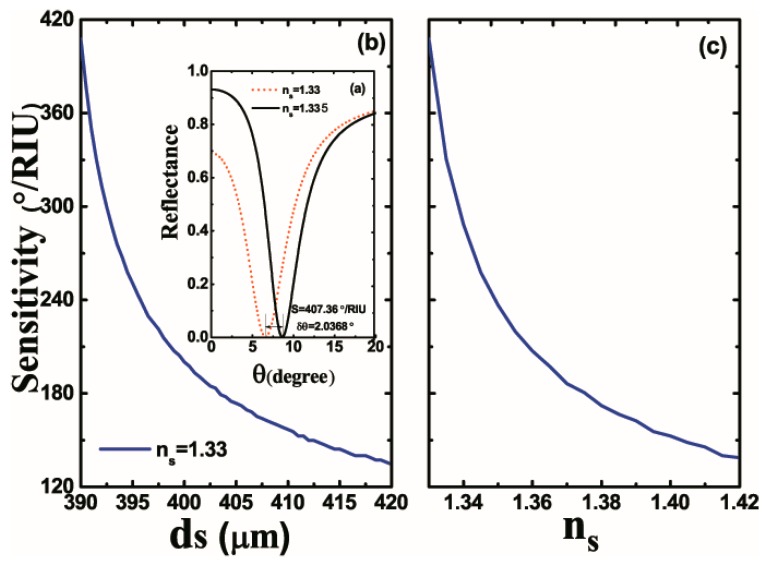
(**a**) Reflectance of the proposed structure with respect to incident angle at $d_{s}=390\mu m$. (**b**) Variations of sensitivity with respect to the thickness of the sensing medium. (**c**) Sensitivity of the proposed structure as a function of the refractive index of the sensing medium.

The thickness of sensing medium is also an important factor in the sensitivity of the proposed structure. Therefore, we plot the curves of sensitivity according to the thickness of the sensing medium, as shown in Figure 6a,b. From the point of view of OTSs mode, it is commonly known that the thickness of the sensing medium is very important to the excitation of OTSs. The thickness of the sensing medium and the excitation of OTSs should satisfy: $d_{s}=d_{0}+m\times \lambda /2n_{b}$, where $d_{0}$ is the minimum value of OTSs observed at λ, and m is a natural number. When $d_{s}=390\;\mu m$, we can get the maximum sensitivity (376.56°/RIU) of the proposed structure. Accordingly, the reflectance as a function of the incident angle is plotted. Since the resonance angle of OTSs excitation is highly sensitive to the thickness of sensing medium, we set $d_{s}$ as 390 μm to ensure higher overall sensitivity and a feasible design of the structure. To conclude, $d_{s}$ is no doubt an important factor in the design of the terahertz refractive index sensor. In order to expand the detection range of the refractive index sensor, we plot a curve to describe the relevance between the sensitivity of refractive index sensor and the refractive index of the sensing medium, as shown in Figure 6c. It is found that the refractive index of the sensing medium shows the same trend as its thickness. Additionally, the sensitivity varies monotonously with $n_{s}$, thus ensuring a large measuring range of $n_{s}$. A sensitivity of 138.59°/RIU can be achieved even if the $n_{s}$ of the sensing medium increases to 1.42.

Therefore, it is proved that proper selection of the sensing medium thickness ($d_{s}$) and the refractive index ($n_{s}$) can produce high sensitivity.

**Figure 7:**
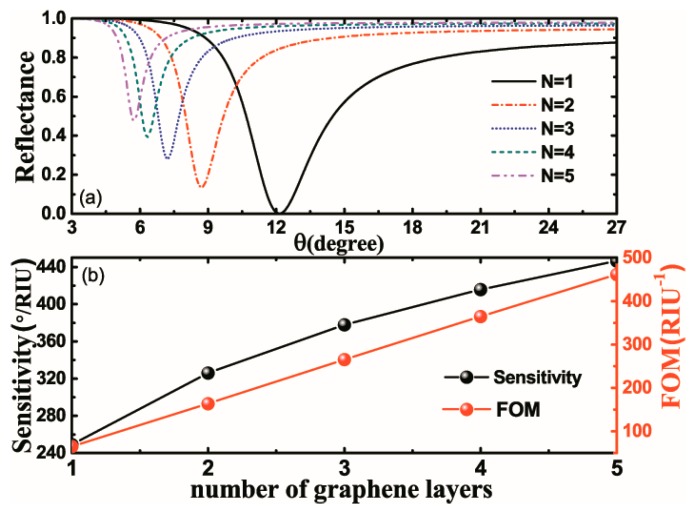
(**a**) Variations of reflectance with respect to the incident angle for different numbers of graphene layers. (**b**) Variations of sensitivity and figure of merit (FOM) of the proposed structure with respect to the number of graphene layers from 1 to 5.

Next, the relationship between the number of graphene layers and the sensitivity of this structure is also investigated, as shown in Figure 7. According to Figure 7a, when the number of graphene layers increases from 1 to 5, the resonance angle tends to shift to a lower position. The variations of sensitivity and FOM as a function of the number of graphene layers are illustrated in Figure 7b. The increase in the number of graphene layers could greatly enhance the local electric field at the interface between the graphene and the sensing medium, which would further change the position of the resonance angle as well as the depth and width of the reflectance, thus improving the sensitivity and FOM. The highest sensitivity (~446.56°/RIU) is obtained when the number of graphene layers increases to 5, and the corresponding FOM would reach 461.59 RIU^−1^. However, it is noteworthy that a continuous increase in the number of graphene layers would lead to a drop in the depth of the reflectance curve, and this would cause difficulties in calculating resonance mode measurements.

We know that numerous approaches to creating a terahertz refractive index sensor have been reported. Lastly, in order to more intuitively reflect the sensing characteristics of the refractive index sensor in this paper, we compiled a table and compared our results with some typical and high-performance previous works, as shown in Table 1. From the table we can see that the main advantages of our design compared to other refractive index sensor are that its sensitivity is higher (although its sensitivity is lower than the grating combining technique scheme of Koju et al. [24], it is at a high level in many recent refractive index-sensitive sensor schemes) and its sensitivity characteristics are much more easily tuned. Also, it needs no phase-matching mechanisms as in the cases of conventional SPR structures. It is also observed that the proposed graphene-covered Bragg reflector structure sensor has a FOM with the same order of magnitude as the majority of schemes reported in the cited references, albeit not as high as structures exhibiting ultra-narrow resonances. Nowadays, the fabrication of 1D PCs and the transfer of graphene are mature technologies, and it is not hard to fabricate the proposed structure as shown in Figure 1. Hence, the proposed structure is a feasible and simple terahertz refractive index sensor method.

## 4. Conclusions

In this paper, we propose a terahertz refractive index sensor based on a graphene–Bragg reflector composite structure and strong resonance mode excitation to provide higher sensitivity at the terahertz band. The findings have shown that the sensitivity and FOM of the proposed structure are heavily dependent on the thickness and refractive index of the sensing medium and the optical parameters of graphene, mainly because these parameters markedly affect the position of the resonance angle as well as the depth and width of the reflectance curve. Proper settings of the parameters could offer a maximal sensitivity of 407.36°/RIU. To our best knowledge, angle-sensitive sensors have rarely been reported in recent years. This sensitivity is at a high level in all kinds of sensor schemes with the same sensitivity expression. This new graphene–Bragg reflector device offers good potential for practical applications in the optical biosensor industry. 

## Figures and Tables

**Table 1:** Comparison between different refractive index sensing methods.

Ref.	Mechanism	Structure	Sensitivity (°/RIU)	FOM	Frequency Range	Tunability
[24]	Bloch surface wave sensor	Grating-photonic crystal structure	2500	/	Visible light	No
[26]	SPR sensor	Kretschmann structure	257	45	Visible light	Yes
[38]	SPR sensor	Grating structure	237	95	Near Infra-red	No
[39]	Waveguide sensor	Silicon waveguide	120	/	Near Infra-red	No
[40]	SPR sensor	Otto structure	34.11	1150	THz	Yes
[41]	SPR sensor	Otto structure	147	/	THz	Yes
[42]	Bloch surface wave sensor	Prism-photonic crystal structure	117	283	THz	Yes
This work	OTSs sensor	Graphene–Bragg reflector structure	400	60	THz	Yes

## References

[1] Buerk D.G. (1995). Biosensors: Theory and Applications.

[2] Piliarik M., Parova L., Homola J. (2009). High-throughput SPR sensor for food safety. Biosens. Bioelectron..

[3] Ashley J., D’Aurelio R., Piekarska M., Temblay J., Pleasants M., Trinh L., Rodgers T., Tothill I. (2018). Development of a β-Lactoglobulin Sensor Based on SPR for Milk Allergens Detection. Biosensors.

[4] Bereza-Malcolm L.T., Mann G., Franks A.E. (2015). Environmental Sensing of Heavy Metals Through Whole Cell Microbial Biosensors: A Synthetic Biology Approach. ACS Synth. Biol..

[5] Ilkhani H., Hughes T., Li J., Zhong C.J., Hepel M. (2016). Nanostructured SERS-electrochemical biosensors for testing of anticancer drug interactions with DNA. Biosens. Bioelectron..

[6] Chung J.W., Kim S.D., Bernhardt R., Pyun J.C. (2005). Application of SPR biosensor for medical diagnostics of human hepatitis B virus (hHBV). Sens. Actuators B Chem..

[7] Ladd J., Taylor A.D., Piliarik M., Homola J., Jiang S. (2009). Label-free detection of cancer biomarker candidates using surface plasmon resonance imaging. Anal. Bioanal. Chemvol..

[8] Conteduca D., Dell’Olio F., Innone F., Ciminelli C., Armenise M.N. (2016). Rigorous design of an ultra-high Q/V photonic/plasmonic cavity to be used in biosensing applications. Opt. Laser Technol..

[9] Ciminelli C., Campanella C.M., Dell’Olio F., Campanella C.E., Armenise M.N. (2013). Label-free optical resonant sensors for biochemical applications. Prog. Quant. Electron..

[10] Farrera C., Andón F.T., Feliu N. (2017). Carbon Nanotubes as Optical Sensors in Biomedicine. ACS Nano.

[11] Konopsky V., Karakouz T., Alieva E., Vicario C., Sekatskii S., Dietler G. (2013). Photonic Crystal Biosensor Based on Optical Surface Waves. Sensors.

[12] Ruan B.X., Guo J., Wu L.M., Zhu J.Q., You Q., Dai X.Y., Xiang Y. (2017). Ultrasensitive Terahertz Biosensors Based on Fano Resonance of a Graphene/Waveguide Hybrid Structure. Sensors.

[13] Shibayama J., Shimizu K., Yamauchi J., Nakano H. (2016). Surface Plasmon Resonance Waveguide Sensor in the Terahertz Regime. J. Lightwave Technol..

[14] Ouyang Q., Zeng S., Jiang L., Hong L., Xu G., Dinh X.-Q. (2016). Sensitivity Enhancement of Transition Metal Dichalcogenides/Silicon Nanostructure-based Surface Plasmon Resonance Biosensor. Sci. Rep..

[15] Shinji H., Dmitry V.N., Zouheir S. (2015). Fano resonance and plasmon-induced transparency in waveguide-coupled surface plasmon resonance sensors. Appl. Phys. Exp..

[16] Mikhailov S.A., Ziegler K. (2007). New electromagnetic mode in graphene. Phys. Rev. Lett..

[17] Li Z.Q., Henriksen E.A., Jiang Z., Hao Z., Martin M.C., Kim P. (2008). Dirac charge dynamics in graphene by infrared spectroscopy. Nat. Phys..

[18] Bonaccorso F., Sun Z., Hasan T., Ferrari A.C. (2010). Graphene photonics and optoelectronics. Nat. Photonics.

[19] Wu L., Chu H.S., Koh W.S., Li E.P. (2010). Highly sensitive graphene biosensors based on surface plasmon resonance. Opt. Express.

[20] Simsek E. (2013). Improving Tuning Range and Sensitivity of Localized SPR Sensors with Graphene. IEEE Photonic Tech. L.

[21] Zhang N.M.Y., Li K., Shum P.P., Yu X., Zeng S., Wu Z. (2017). Hybrid Graphene/Gold Plasmonic Fiber-Optic Biosensor. Adv. Mater. Technol..

[22] Rodrigo D., Limaj O., Janner D., Etezadi D., de Abajo F.J.G., Pruneri V. (2015). Mid-infrared plasmonic biosensing with graphene. Science.

[23] Xu S., Zhan J., Man B., Jiang S., Yue W., Gao S. (2017). Real-time reliable determination of binding kinetics of DNA hybridization using a multi-channel graphene biosensor. Nat. Commun..

[24] Koju V., Robertson W.M. (2017). Leaky Bloch-like surface waves in the radiation-continuum for sensitivity enhanced biosensors via azimuthal interrogation. Sci. Rep..

[25] Sun Y., Zeng W., Sun H., Luo S., Chen D., Chan V. (2018). Inorganic/polymer-graphene hybrid gel as versatile electrochemical platform for electrochemical capacitor and biosensor. Carbon.

[26] Sun P., Wang M., Liu L., Jiao L., Du W., Xia F. (2019). Sensitivity enhancement of surface plasmon resonance biosensor based on graphene and barium titanate layers. Appl. Surf. Sci..

[27] Deng X., Tang H., Jiang J. (2014). Recent progress in graphene-material-based optical sensors. Anal. Bioanal. Chem..

[28] Xiao S., Zhu X., Li B.-H., Mortensen N.A. (2016). Graphene-plasmon polaritons: From fundamental properties to potential applications. Front. Phys..

[29] Zhao Y., Jiang J. (2018). Recent Progress on Neuromorphic Synapse Electronics: From Emerging Materials, Devices, to Neural Networks. J. Nanosci. Nanotechnol..

[30] Li N., Tang T., Li J., Luo L., Sun P., Yao J. (2018). Highly sensitive sensors of fluid detection based on magneto-optical optical Tamm state. Sens. Actuators B Chem..

[31] Tsurimaki Y., Tong J.K., Boriskin V.N., Semenov A., Ayzatsky M.I., Machekhin Y.P. (2018). Topological Engineering of Interfacial Optical Tamm States for Highly Sensitive Near-Singular-Phase Optical Detection. ACS Photonics.

[32] Brand S., Kaliteevski M.A., Abram R.A. (2009). Optical Tamm states above the bulk plasma frequency at a Bragg stack/metal interface. Phys. Rev. B.

[33] Zhang W.L., Wang F., Rao Y.J., Jiang Y. (2014). Novel sensing concept based on optical Tamm plasmon. Opt. Express.

[34] Ye Y., Xie M., Tang J., Ouyang J. (2019). Highly sensitive and tunable terahertz biosensor based on optical Tamm states in graphene-based Bragg reflector. Results Phys..

[35] Zhan T., Shi X., Dai Y., Liu X., Zi J. (2013). Transfer matrix method for optics in graphene layers. J. Phys. Condens. Matter.

[36] Jepsen P.U., Jensen J.K., Møller U. (2008). Characterization of aqueous alcohol solutions in bottles with THz reflection spectroscopy. Opt. Express.

[37] Su Y. (2014). Investigation of liquid monohydric alcohols by Terahertz time-domain spectroscopy. Opt. Instrum..

[38] Cai D., Lu Y., Lin K., Wang P., Ming H. (2008). Improving the sensitivity of SPR sensors based on gratings by double-dips method (DDM). Opt. Express..

[39] Jiao Y., Wess S.M. (2010). Design parameters and sensitivity analysis of polymer-cladded porous silicon waveguides for small molecule detection. Biosens. Bioelectron..

[40] Purkayastha A., Srivastava T., Jha R. (2016). Ultrasensitive THz–Plasmonics gaseous sensor using doped graphene. Sens. Actuat. B: Chem.

[41] Xiang Y., Wu J.Z.L., You Q., Ruan B., Dai X. (2018). Highly Sensitive Terahertz Gas Sensor Based on Surface Plasmon Resonance with Graphene. IEEE Photonics J..

[42] Baghbadorani H.K., Barvestani J., Entezar S.R. (2017). Biosensors based on Bloch surface waves in one-dimensional photonic crystal with graphene nanolayers. Appl. Optics.

